# A239 PERFORMANCE OF CLINICAL RISK PREDICTION MODELS FOR POST-ERCP PANCREATITIS: A SYSTEMATIC REVIEW

**DOI:** 10.1093/jcag/gwae059.239

**Published:** 2025-02-10

**Authors:** N Sabrie, G Minahs, M Vaska, Z W Meng, D Brenner, N Forbes

**Affiliations:** University of Toronto, Toronto, ON, Canada; McMaster University, Hamilton, ON, Canada; University of Calgary, Calgary, AB, Canada; University of Calgary, Calgary, AB, Canada; University of Calgary, Calgary, AB, Canada; University of Calgary, Calgary, AB, Canada

## Abstract

**Background:**

Pancreatitis is common following endoscopic retrograde cholangiopancreatography (ERCP). Despite increased vigilance of post-ERCP pancreatitis (PEP), both its incidence and associated mortality are rising. Risk prediction models may provide more accurate stratification of patient risk and proactive mitigation of PEP incidence and/or severe associated outcomes.

**Aims:**

We aimed to systematically review and summarize the available literature on PEP risk prediction models and their respective peformance.

**Methods:**

We conducted an electronic search of MEDLINE, PubMEd, Cochrane, and CINAHL from inception through April 9, 2024 for studies evaluating the details and performances of available PEP prediction models. Studies were eligible if they used statistical measures to quantify their model’s predictive ability. Risk of bias was determined using the PROBAST tool.

**Results:**

Nineteen studies met eligibility criteria and were included. Logistic regression models were used in 15 studies, with machine learning models representing the second most commonly employed approach. Ten studies reported the performance of their risk prediction models using derivation data, with areas under the receiver operating curve (AUC) ranging from 0.68 to 0.86. Fifteen studies reported the performance of their risk prediction models on internally validated data, with AUCs ranging from 0.66 to 0.97. Eight studies reported on the performance of their risk prediction models on external validation data, with AUCs ranging from 0.67 to 0.98.

**Conclusions:**

Numerous PEP clinical prediction models exist with variable performances. The use of PEP prediction tools can support the management of patients following ERCP. Implementation studies assessing the optimal usability of these tools, followed by prospective evaluations, are needed to evaluate their potential impacts on reducing PEP in real-world practice.

Table 2. Summary of methods used to inform post-ERCP pancreatitis prediction models.

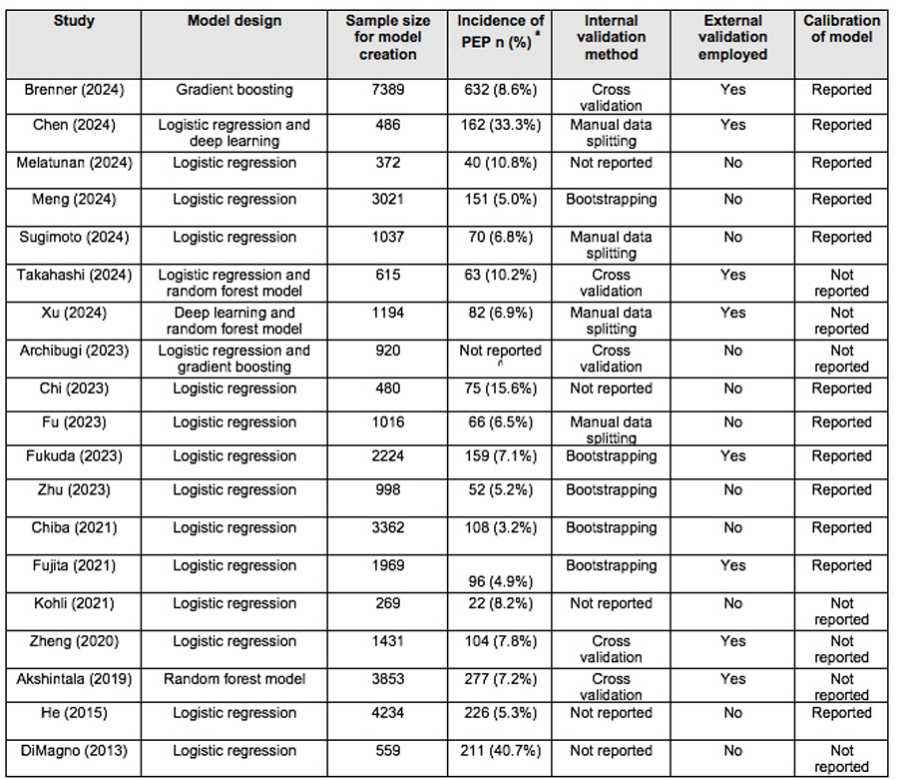

**Funding Agencies:**

None

